# *An Account of the Experiments and Discoveries of* Lewis Galvani, *Professor of Anatomy at Bologna, Relative to the Powers of Electricity in Muscular Motion*

**Published:** 1792

**Authors:** 


XVIII. *An Account of the Experiments and Difcoveries of* Lewis Galvani, *Profeffor of Anatomy at Bologna, relative to the Powers of Electricity in Mufcular Motion.—Vide* Aloyfii Galvani *de Viribus Electricitatis in Motu Mufculari Commentarium.* 4to. Bologna, 1791.

FROM a fact mentioned in a periodical work, publifhed in the year 1786 at Bologna, relative to a Student of Phyfic, who, in diffecting a moufe alive, was much furprifed, on touching the intercoftal nerve with his fcalpel, to experience an electric fhock of confiderable ftrength in his hand, the Abbé Vaffalli, Profeffor of Natural Philofophy at Tortona, conjectured that Nature had fome means of accumulating and retaining electricity in fome parts of an animal body, and of making ufe of it occafionally. He was confirmed in this opinion by fome experiments, an account of which he publifhed in 1789; but Profeffor Galvani, (to whom the Public are already indebted for feveral valuable communications* on different fubjects of Anatomy) led on by an accidental circumftance, has gone much farther, and has

* *Vide* De Bonon. Inftit. Comment. Tom. V. et VI.

opened opened to physiologists a source of new ideas relative to muscular motion.

As he was dissecting a frog in a room in which some of his friends were amusing themselves with electrical experiments, at the very instant he happened to touch a nerve of the frog with his knife some one drew a spark from a chain connected with the electrical apparatus, and the body of the animal immediately became convulsed.

The Professor, attributing this effect to his having accidentally wounded the nerve, pricked it in reality, but excited no motion. He then touched it at the moment another spark was drawn from the electric chain, and the contraction again took place. He made the same experiment a third time, but without any such effect. He now perceived that he held his knife by the handle, which was of bone, and, of course, a bad conductor. He repeated the experiment several times with different non-conducting substances, and no contraction ensued; but it constantly took place whenever metallic bodies were applied to the nerves.

He now fastened to a nerve an iron wire of considerable length, and upon a spark being drawn from the electric chain the convulsive motions were renewed: he therefore called this wire the *nervous conductor*.

Inftead of a wire he afterwards fubftituted an iron hook fixed to the fpinal marrow of the frog. In fome of his experiments he brought the frog itfelf near to the electrical machine, and in others the conductor alone, the animal itfelf remaining at a confiderable diftance, and in either way contractions were always produced. They were even obtained, we are told, by an infulated conductor of more than two hundred feet long. They were found to be ftronger if, by means of a conducting body, the feet of the animal were made to communicate with the earth; and under thefe circumftances the phenomenon, it feems, was conftant, whether the animal was infulated or not.

The effect produced by conducting bodies, which communicated with the feet of the animal, led him to fufpect that conducting bodies applied to mufcles might occafion this contractile motion. He therefore faftened to a mufcle metallic wires, which he diftinguifhes by the name of *mufcular conductors*, but without effect, for, if the nervous conductor were wanting, no motion could be excited.

A frog, prepared for the experiment by removing its integuments, was laid on a non-conducting furface, on which the nervous conductor was fo placed as to be at the diftance of feveral

ſeveral lines, or even an inch from the nerve. The moment a ſpark was drawn from the electrical apparatus the limbs of the animal contracted. The ſame thing happened, we are told, on placing the animal on a conducting ſurface, and the nerves with their conductor on a non-conducting ſurface. No difference was obſerved when the nervous conductor was covered through its whole length with ſealing wax.

The animal was afterwards placed on the magic picture, and a ſtrong ſpark was drawn, but without exciting any contraction.

All theſe experiments having been made with poſitive electricity, the Profeſſor was deſirous of ſeeing if the ſame effects would be produced by negative electricity.

With this view he inſulated a frog, prepared as in the former experiment, and a man. The latter drew ſparks from the ſurrounding bodies, and this produced the ſame effects as before on the animal. The ſame thing happened when he made the nervous conductors communicate with the negative ſurface of a Leyden phial, in whatever way the ſparks were drawn. He experienced the ſame effects, 1. with the electrophorus upon bringing the animal near the electrometer; 2. on placing the animals in veſſels, at a great diſtance, by means of nervous conductors connected with the nerves that are diftributed to the furface of the body.

He feparated the crural nerves of a frog from the furrounding parts, and having removed them from the mufcle, he applied the conductor to the latter, and when fparks were drawn from the electrical apparatus a motion was excited in the correfponding limb of the animal.

In all thefe experiments the animals had communicated, by means of the furrounding air, with the electrical apparatus. He therefore tried if there would be any difference in the refult by interrupting this communication, or fuppreffing it altogether.

For this purpofe the animal was placed under a glafs veffel, and upon fparks being drawn was found to contract as before. Other veffels were placed over this, and the fame contractions were obferved to be weaker and weaker in proportion to the number and thicknefs of the veffels. The animal was even placed under the receiver of an air pump, and whether the air was drawn out or not, at the moment fparks were drawn, fome degree of contraction was obfervable.

When the nervous conductors were brought near the electric chain, though no fpark was drawn, the animal was ftrongly agitated.

Hitherto the experiments had been made on animals of cold blood. He now repeated them on fowls and ſheep; the reſults were the ſame; and from theſe experiments he is led to conclude, that the animals the beſt calculated for them are the older ones, and thoſe whoſe muſcles are the whiteſt. He has found that the fleſh of animals which have been ſubject to theſe trials corrupts ſooner than that of others.

After numerous experiments with artificial electricity, he was induced to have recourſe to natural electricity, drawn from the atmoſphere by means of a conductor fixed to the top of his houſe, and communicating with his chamber by a metallic wire connected with it. On this wire he ſuſpended animals of cold blood, and others, properly prepared, and by means of wires faſtened to their legs formed a communication between them and the ground. Every time there was lightning the animals were affected with ſtrong contractile motions, which preceded, and were obſerved to correſpond with the intenſity and frequency of the thunder. Even when there was no lightning ſimilar movements were excited as often as ſtormy clouds paſſed over the houſe; but when there was lightning, lightning, with a serene sky, the animals exhibited no appearance of contraction.

In these different experiments the Professor had considered only the electricity which is extraneous, as it were, to the bodies of animals; but in the course of his inquiries an accidental circumstance directed his attention to the electricity which is peculiar to, and inherent in, animals.

He had suspended, by means of metallic hooks fixed to their spinal marrow, several frogs on an iron balcony in his garden, and had repeatedly observed that these animals gave signs of contraction. At first he thought this might be owing to some changes in the atmosphere: but a more accurate inquiry convinced him he was mistaken; for having placed in his chamber, on an iron plate, an animal, properly prepared, with the hooks fixed to its spine, on pressing it against the plate he saw, with surprise, the same motions he had observed in the animals suspended on the balcony. Making use of different metals, he tried them at different times, and on different days, and always with the same results, except that the contractions varied according to the diversity of the metals. He found that silver was better than any of the others for thefe purpofes. He made fimilar experiments with non-conducting bodies, but always without fuccefs. Hence he began to fufpect that the animal had really an electricity peculiar to itfelf. This fufpicion was confirmed when he found that the circulation of the nervous fluid from the nerves to the mufcles, at the time the phenomenon happens, is nearly fimilar to the circulation of artificial electricity in the Leyden phial. The following fact led him to this difcovery:

While he was holding with one hand, by means of a hook, a prepared frog, in fuch a manner that its feet touched a fmall filver bafon, he happened accidentally with his other hand to touch the bafon; violent contractions immediately took place in the whole body of the animal, which were renewed every time the fame mode of communication was repeated. When one perfon held the frog, and another touched the difh, the animal remained immoveable.

Having thus perceived the neceffity of a communication to excite motion, he engaged in frefh experiments on this fubject. He placed, on a non-conducting furface, a prepared frog, and applied one end of a bent wire to the hook connected with the nerve, and the other end to the the feet, or to the mufcles of the legs of the animal, and there was immediately a contraction. If the bent wire was interrupted by a non-conducting fubftance, the frog remained motionlefs.

When a frog, properly prepared, was fufpended by the extremity of the foot, if the hook touched a metallic plate, and at the fame time the other leg of the animal touched this plate, this leg immediately contracted.

The Profeffor found that different metals were productive of different effects in thefe experiments. If the plate, the hook, and the bent wire, were all of iron, the motion was more feeble, or even altogether wanting; but if one of thefe was of iron, and the reft were of copper, or, what anfwered ftill better, of filver, the contraction immediately took place, and continued a much longer time.

As the circulation of the Leyden phial fuppofes two contrary electricities, the one more condenfed, or pofitive, and the other lefs fo, or negative, fo Profeffor Galvani concludes, that a fimilar diftinction takes place in the bodies of animals, and that one of thefe electricities, viz. the condenfed, or pofitive, is feated in the nerves, and the other in the mufcles.

He

He was led to thefe conclufions by the following experiments :

He applied, in fome inftances, glafs cylinders, and in others fealing wax, to the fpinal marrow of frogs: with the former he obtained no motion in the animal, but the latter conftantly excited it. If the fpine of the frog was covered with tin foil, the fealing wax, though at the diftance of the third part of an inch, or more, excited mufcular contraction; and upon bringing the frog near the electrical machine, and turning the latter feveral times, the animal exhibited no fign of motion. This proves, he thinks, that the electricity of the nerves is pofitive.

He made, in the fame manner, experiments to afcertain if it were poffible to excite motion in the mufcles; but in thefe no fuch effect took place.

He next coated a nerve with tin foil, and obtained ftrong movements upon touching this coating with different bodies. Similar effects took place when the brain was coated in like manner; but when the mufcles were coated, inftead of the nerves, the animal remained immoveable, or afforded only very feeble figns of contraction.

Profeffor

Profeffor Galvani endeavoured to afcertain whether the electricity is propagated from the nerve through the whole nervous fyftem, or is confined to the nerve which is the fubject of the experiment; and he found that when the nerve is not feparated, but only laid bare, the electricity fpreads itfelf through the whole body.

To prove that thefe phænomena are really the effects of electricity, he had recourfe to Dr. Franklin's magic picture, fo that the nerves touched one of its furfaces, and the mufcles the other, and upon applying the conductor a very fenfible contraction took place.

